# Prevalence and risk factors of psychiatric disorders in an industrial population in India

**DOI:** 10.4103/0019-5545.33256

**Published:** 2007

**Authors:** Srihari Dutta, Nilamadhab Kar, Jagadisha Thirthalli, Sreekumaran Nair

**Affiliations:** State Office, UN House - 1, Surya Nagar, Bhubaneswar, Orissa, India; * Wolverhampton City Primary Care Trust; Wolverhampton, UK; ** National Institute of Mental Health and Neurosciences, Bangalore, India; *** Kasturba Medical College, Manipal, India

**Keywords:** Industry, prevalence, psychiatric disorder, risk

## Abstract

**Background:**

Recent information on psychiatric morbidity in industrial employees is not available in India. Such information may help in building mental health care for this population.

**Aim:**

The aim was to study the prevalence of psychiatric morbidity and the risk factors associated with it in an industrial population.

**Materials and Methods:**

Two hundred thirty-eight individuals were selected by a stratified randomisation technique and screened using the General Health Questionnaire-12 (GHQ-12), Johns Hopkins University Hospital Test for alcoholism and a semistructured questionnaire for other substance use, sleep problems and past psychiatric history. Following a detailed clinical interview, diagnoses were based on International Classification of Diseases (ICD)–10, Classification of Mental and Behavioural Disorders: Diagnostic Criteria for Research (DCR).

**Results:**

The prevalence rate for psychiatric disorder of one month's duration in the study population was 51.7%. Substance use, depression, anxiety and sleep disorders were common. Comorbidities were found in 65% of the subjects. Both univariate analysis and stepwise multiple regression revealed that educational level, perceived stress, job satisfaction and stressful life events were the independent determinants of psychiatric morbidity.

**Conclusion:**

A significant proportion of industrial employees had psychiatric morbidity and many psychosocial factors were associated with caseness.

Work environments are known to influence the psychological functioning of the individual.[1,2] In comparison with the general population, industrial workers have the added risk of physical, chemical, biological and other specific psychosocial factors of their occupational environment.[3] In addition, there are indications that modern mechanisms of production and methods of trade are contributing to an increase in stress risks in industry.[4] Psychiatric disorders constitute the leading occupational health problems with one-third of all workers reporting adverse psychological effects.[5]

About 15% of all occupational disabilities are stress related.[6] Minor psychiatric morbidity is the most common cause for sick leave in industrial occupations.[7] Compensation claims for all stress-related disorders are growing in number while all other disabling work injuries are decreasing.[8] Epidemiological studies can explore the relationship between psychiatric morbidity and work environment and their findings may help in prevention of such morbidity.[9]

The reported prevalence rates of psychiatric morbidity in the Indian industrial population range from 14-37% and can be up to as high as 74% in Western reports.[10–12] Most of the prevalence studies in industry noted above were conducted without the use of any specific diagnostic criteria for psychiatric disorders. Prevalence rates for psychiatric disorders according to epidemiological studies in the general population in India varied from 0.95-13% (estimated prevalence rate of 5.82% was found for 13 Indian epidemiological studies).[13]

This indicates that the reported industrial rates of psychiatric morbidity have always been higher than that of the general population. Rapid changes in illness status are observed in the industrial population[14] due to the various influencing factors.[15] These issues undermine periodic evaluation of mental health status and needs of the industrial employees, which are designed to help develop mental healthcare in industrial set-ups. The specific aims of this study were to determine the prevalence of psychiatric disorders in an industrial set-up and to study the factors associated with the morbidity.

## MATERIALS AND METHODS

The study was conducted in a chemical fertilizer company. All the permanent employees enrolled by the company (*n* = 780) were considered as the universe for the study. The employees were stratified according to the work-type, namely, executives (*n* = 248, 31.8%) and nonexecutives (*n* = 532, 68.2%); and the samples were selected proportionately from both the groups. With an expected average prevalence of 25% and with an allowable error of 5%, the sample size was estimated to be 210 at a 95% confidence interval (CI). By giving 10% for nonresponse error, the final sample size obtained was 231.

We randomly selected a total of 238 employees including 77 (31.0%) executives and 161 (30.3%) nonexecutives for the study. The interviews of the selected employees were conducted on three specified days in a week, in a secluded room at the site of work of the employees to ensure privacy. Written informed consent was taken from all the subjects and confidentiality was assured. All the persons were interviewed individually and there was no nonresponse. The evaluation of the subjects was done in two phases consisting of a screening phase and a diagnostic phase.

In the screening phase, socio-demographic data including age, education, family type, current living arrangements and permanent residence were collected in a semistructured proforma. Information on job attributes like type of work, years of industrial experience, rotating shift work were obtained. The participants were asked to reflect their global impressions on job satisfaction, job stress, interpersonal relationships in the workplace and perceived family support as positive or negative. The physical diagnoses for which they were being treated were also noted. Stressful life events experienced by the employees in the previous one year period were assessed using the presumptive stressful life event scale (PSLES).[16]

The subjects were screened by the General Health Questionnaire (GHQ-12),[17] which is a widely utilized screening instrument for epidemiological studies and has been proved to be useful[18] and highly discriminatory.[19] Semistructured proformas were used to screen for data on substance use and shift work-related sleep problems. The Johns Hopkins University Hospital Test[20] was used to screen for alcoholism.

A person was considered to be screen-positive if he was found to be having any of the following conditions: (i) a score of two or more on GHQ-12; (ii) probable substance use disorders or a score of one or more in the Johns Hopkins University Hospital Test for screening of alcoholism; (iii) sleep problems and (iv) past history of psychiatric disorder. All those who were considered as screen-positive underwent a detailed clinical psychiatric interview following a semistructured proforma. The clinical profile of each subject was prepared considering the findings of the screening and the detailed clinical interview.

These profiles were independently analysed by two psychiatrists (NK and J) and diagnoses were made according to ICD criteria (ICD-10-DCR).[21] The evaluators made same the diagnoses for 70.9% of the whole sample. The cases for which the evaluators had different diagnoses were discussed in detail and consensus regarding the diagnoses was reached.

### Statistical analysis

Prevalence was calculated in terms of percentage and its 95% CL. Univariate analysis was carried out initially to identify risk factors. These results were expressed in terms of unadjusted odds ratios (OR), their 95% CIs and *P* values. Furthermore, stepwise multiple logistic regression analysis was carried out to identify independent predictors. These results were expressed in terms of adjusted OR and their 95% CIs. The level of significance was set at a standard of 0.05.

## RESULTS

The sample had an age range of 22-56 years with a mean of 34.4 ± 6.37 (mean ± standard deviation) years. The average years of industrial work experience was 11.99 ± 5.94 years (range = 1-29). There were 51 (21.4%) engineers, 14 (5.9%) technical administrators and 12 (5.0%) nontechnical administrators, who form the executive group. The nonexecutive group consisted of 43 (18.0%) semiskilled workers, 88 (36.97%) technicians and 30 (12.6%) clerks.

After the screening phase, 103 persons were found to be GHQ-positive, 142 persons had probable pathological substance use problems, 69 persons had sleep problems and 30 persons had past psychiatric history.

In total, 200 persons were found to be screen-positive (some persons were screen-positive by more than one screening method) and were selected for a detailed clinical interview. The prevalence of different psychiatric disorders is given in Table 1. Almost half (*n* = 123, 51.7%) had some psychiatric diagnosis. It was observed that harmful use or dependence on tobacco was the sole diagnosis in 17 (7.14%) persons. Substance use disorders were comorbid conditions in all other subjects except two (one with harmful use of alcohol and the other with cannabis).

**Table 1:** Prevalence of different psychiatric disorders

Diagnoses	*n*	Percentage	95% CI
Alcohol harmful use / dependence	16	6.7	3.52-9.88
Cannabis harmful use	7	2.94	0.79-5.09
Sedatives or hypnotics harmful use	3	1.26	-0.15-2.68
Opium dependence	1	0.42	-0.40-1.24
Caffeine harmful use / dependence	7	2.94	0.79-5.09
Tobacco harmful use / dependence	68	28.57	22.83-34.31
Schizophrenia	1	0.42	-0.40-1.24
Nonorganic psychosis unspecified	1	0.42	-0.40-1.24
Depressive episodes	38	15.96	11.31-20.61
Dysthymia	16	6.72	3.54-9.90
Panic disorder/GAD/MADD/anxiety unspecified	18	7.56	4.20-10.91
Phobic disorders	14	5.88	2.89-8.87
Obsessive compulsive disorder	14	5.88	2.89-8.87
Adjustment disorders	6	2.52	0.53-4.51
Dissociative disorder	1	0.42	-0.40-1.24
Somatoform disorders	3	1.26	-0.16-2.68
Other neurotic disorders	2	0.84	-0.31-2.00
Sleep disorder	31	13.02	8.74-17.30
Sexual dysfunction	1	0.42	-0.40-1.24
Unspecified mental disorder	1	0.42	-0.40-1.24
Any diagnosis	123	51.67	45.32-58.02

GAD: Generalised anxiety disorder; MADD: Mixed anxiety depressive disorder; Numbers (*n*) add up to more than 100% as a single person may have more than one diagnosis

Most (*n* = 80, 65.0%) of the diagnosed persons had comorbidities. Out of the 123 who had psychiatric diagnoses, 43 (34.9%) persons had one, 48 (39.0%) had two and 32 (26.0%) had three or more diagnoses. More common comorbid diagnoses were harmful use or dependence on nicotine (39.02%), sleep disorder (13.0%), phobic disorders (11.38%), harmful use or dependence on alcohol (7.31%), harmful caffeine use (5.69%), obsessive-compulsive disorder (5.69%), depression (4.06%) and dysthymia (4.06%).

The common physical diagnoses were acid peptic disease (33, 13.86%), headache (20, 8.40%), hypertension (15, 6.30%) diabetes mellitus (11, 4.62%) and asthma (8, 3.36%). Demographic factors associated with psychiatric caseness are given in Table 2. Comparing noncases (*n* = 115) with cases (*n* = 123) showed no differences in the their mean ages (noncase = 34.26 ± 6.17 *vs* case = 34.48 ± 6.58; t = - 0.264, *P* = 0.792), work experience in years (noncase = 12.19 ± 5.97 *vs* case = 11.79 ± 5.93; t = 0.516, *P* = 0.607); or stress levels according to PSLES in the previous year (noncase: *n* = 8, 167.62 ± 77.62 *vs* case *n* = 35, 174.11 ± 102.03; t = − 0.168, *P* = 0.867). However, a significant difference was noted in the incomes of the two groups. Cases had significantly lower incomes compared to the noncase group (noncase 6.176 ± 2.645 *vs* case 5.03 ± 3.29 × 1000 Rs. Per month; t = 2.934, *P* = 0.004). Work-related attributes associated with psychiatric morbidity are given in Table 3.

**Table 2:** Demographic associates of psychiatric morbidity: univariate analysis

Variable		Total screened *n* (%)	Prevalence *n* (%)	Odds ratio	95% CI
Age groups (years)	≤ 30*	75 (31.5)	38 (50.7)		
	31-40	127 (53.4)	67 (52.8)	1.01	0.54-1.86
	> 40	36 (15.1)	18 (50.0)	0.97	0.41-2.32
Education	School*	99 (41.6)	61 (61.6)		
	College / technical diploma	70 (29.4)	34 (48.6)	0.59	0.30-1.14
	University / technical degree	69 (29.0)	28 (40.6)	0.43	0.22-0.84
Income in rupees per month	< 3500*	48 (20.2)	36 (75.0)		
	3501-6000	114 (47.9)	54 (47.4)	0.30	0.13-0.67
	6001-8500	49 (20.6)	23 (46.9)	0.29	0.11-0.76
	> 8501	27 (11.3)	10 (37.0)	0.66	0.23-1.93
Marital status	Ever married*	183 (76.9)	90 (49.2)		
	Never married*	55 (23.1)	33 (60.0)	1.55	0.81-2.99
Family	Joint*	38 (16.0)	21 (55.3)		
	Nuclear	145 (60.9)	69 (47.6)	0.73	0.34-1.60
	Living alone	55 (33.1)	33 (60)	1.21	0.48-3.05
Living with family	Yes*	157 (66.0)	74 (47.1)		
	No	81 (34.0)	49 (60.5)	1.72	0.96-3.07
Immigration status	Local*	188 (79.0)	103 (54.8)		
	Immigrant	50 (21.0)	20 (40.0)	0.55	0.28-1.09

* Reference group

**Table 3:** Univariate analysis of the association between work-related attributes and psychiatric morbidity

Variables		Total screened (*n* - 238) *n* (%)	Proportion of cases (*n* = 123) *n* (%)	Statistics	
					
				Odds ratio	95% CI
Job	Executive	77 (32.4)	33 (42.9)	0.59	0.33–1.06
	Non-executive*	161 (67.6)	90 (55.9)		
Shift	General*	122 (51.3)	61 (50.0)	1.15	0.67–1.97
	Rotating	116 (48.7)	62 (53.4)		
Perceived support	Yes*	200 (84.0)	98 (49.0)		
	No	38 (16.0)	25 (65.8)	2.0	0.92–4.41
Perceived stress	Yes	100 (42.0)	60 (60.0)	1.79	1.03–3.11
	No*	138 (58.0)	63(45.7)		
Job satisfaction	Yes*	143 (60.1)	62 (43.4)		
	No	95 (29.9)	61 (64.2)	2.34	1.33–4.15
Interpersonal relationship	Satisfactory*	214 (89.9)	107 (50)		
	Not satisfactory	24 (10.1)	16 (66.7)	2.00	0.77–5.35
Life Event	Absent*	195 (81.9)	88 (45.1)		
	Present	43 (18.1)	35 (81.4)	5.32	2.22–13.16
Physical illness	Absent*	159 (66.8)	76 (47.8)		
	Present	79 (33.2)	47 (59.5)	1.60	0.92–2.88
Past psychiatric history	Absent*	208 (87.4)	103 (49.5)
	Present	30 (12.6)	20 (66.7)	2.04	0.86–4.94

* Reference group

Semiskilled workers had more psychiatric morbidity than executives (OR: 3.88, CI: 1.59-9.60). Univariate analysis shows that education, income, perceived stress, job satisfaction and stressful life events were the significant determinants of psychiatric morbidity. Results of stepwise multiple regression are given in Table 4.

**Table 4:** Risk factors associated with psychiatric morbidity: Results of stepwise multiple logistic regression analysis

Factors	Categories	Adjusted OR	95% CI
Education	School*	1.00	
	College	0.48	0.24–0.95
	University	0.27	0.13–0.57
Perceived stress	No*	1.0	
	Yes	2.31	1.25–4.25
Job satisfaction	Yes*	1.0
	No	2.09	1.14–3.83
Interpersonal	Satisfactory*	1.00	
relationship	Unsatisfactory	2.22	0.79–6.28
Life events	Absent*	1.00	
	Present	4.32	1.80–10.35

* Reference group

Shift duties were associated with more sleep disorders. We found that 26 (83.9%) out of 31 persons having sleep disorders had shift duties (χ^2^ =17.6, *P* = 0.000). Examining the different life events experienced by persons with psychiatric disorders, the common ones were lack of a child (12.19%), death of a close family member (9.75%), marital separation (9.75%), suspension from job (9.75%), extramarital relationship of the spouse (7.3%), death of spouse (5.9%), property damage (4.87%) and death of a friend (4.06%).

## DISCUSSION

The study sample was selected using a stratified random sampling technique. Four different case-finding methods were used to detect probable cases in the screening phase. ICD-10-DCR[21] criteria for diagnoses were used and the clinical profile of each subject was evaluated separately by two psychiatrists and the evaluations were found to have good concordance. The prevalence figures for psychiatric morbidity in an industrial set-up observed in the index study are substantially higher in comparison with those from general population figures from India.[13]

This suggests that industrial populations are at a greater risk of developing psychiatric morbidities. However, the comparison should be viewed with caution as the studies differ in their diagnoses, age groups considered and the diagnostic methods used. Both higher and lower rates of prevalence of psychiatric morbidity have been reported in industrial set-ups elsewhere.[5,11,14] However, we found higher morbidity in the index study than the reported figures in industrial set-ups from India.[10–12] The higher overall prevalence rates in the index study may be due to the use of extensive screening methods and research criteria for diagnostic processes as well as the evaluation for a wider range of psychiatric disorders as compared to previous studies.

Prevalence figures determined by these methods are expected to be more accurate. However, higher prevalence could also be due to factors like the type of industry being studied. Thus, caution must be exercised in comparing studies in different settings. This finding also generates a hypothesis as to whether psychiatric morbidity is different in different industries. The overall prevalence rates for psychiatric disorders had gone up by at least 7.14% due to harmful use or dependence on tobacco as a sole diagnosis. However, the relevance of such use or dependence is more of a public health concern than a psychiatric disorder. The substance ‘harmful use’ or ‘dependence’ rates in the index study are higher than those reported in the general Indian population.[13]

Psychiatric disorders, which were most prevalent in the industrial population under study, were depression, anxiety and sleep disorders. Depressive episodes and dysthymia were present in more than one in five cases. There was an association between lack of job satisfaction and perception of stress, which could lead to depressive states. Panic, phobic and obsessive-compulsive disorders were more common among anxiety disorders. Perceived stress in the environment might be related to the higher prevalence of anxiety disorders but the exact relationship requires further study.

Sleep disorders found in a sizeable proportion of the study population, were found to be significantly associated with shift duty. It has been documented that shift work leads to sleep disturbance and this in turn affects health and well-being,[22] results in more mistakes due to concentration impairment and makes the worker more vulnerable to accidents.[23] Understandably, sleep disorders have relatively greater importance for an industrial worker.

Comorbidities were common in persons having psychiatric problems in this index study. More frequent diagnoses were substance use, sleep, phobic and depressive disorders. It is apparent that most of the physical comorbidities seen in these cases are known psychosomatic disorders and are thus influenced by psychological factors.

### Risk factors

There was no influence of age, marital status, family type or presence of physical illness on the overall psychiatric morbidity of a person in the index study. A low level of education was associated with increased psychiatric morbidity, which could be due to an indirect association with the type of work and lower income. In comparison with the executives, a greater proportion of semiskilled workers had higher morbidity. The incidence of psychiatric morbidity was found to decrease with increase in income levels, which was consistent with previous findings.[24]

The prevalence of psychiatric morbidity was significantly high among employees who perceived stress as compared to those who did not. Psychiatric symptoms were known to be the result of increased stress levels at work,[5] dysfunctional interpersonal relationships,[25] increased job pressure, greater responsibility without authority, feeling of insecurity,[6] career problems and pressure for production.[4] On the other hand, stress perception can be the function of a depressed mood with higher proportions of perceived stress due to unsuitable jobs and human relations reported in depressives than in the controls in industry.[26]

These findings suggest that perceived stress can be both the cause and effect of psychiatric disorders. In the index study, those who did not have job satisfaction were found to be at greater risk for psychiatric disorders (OR: 2.34; CI: 1.33-4.15). Poor job satisfaction may itself be a form of continuing stress and may also stem from various factors like poor interpersonal relationships in the workplace. Interpersonal conflict at work and psychiatric morbidity were reported to be significantly related.[27] Although not statistically significant, 66% of employees who reported poor interpersonal relationship in the work place in the index study, had psychiatric morbidity.

### Life events

The presence of a life event was found to be a significant determinant for psychiatric morbidity in our study with 28.4% of the persons with psychiatric disorders reporting stressful events, which was significantly greater than the number of normal persons who reported stressful events (6.95%). However, the severity of the events was not significantly different in both the groups. While these stresses might have contributed to the morbidity, it is difficult to assume a causal relationship as morbidity is influenced by various factors. It has been reported that besides causing illness, psychiatric morbidity may also produce new events[28] such as an inability to work, loss of job, worsening of relationships, financial problems and increasing the likelihood to experience interpersonal conflict at work.[27]

In addition, the nature of an event, its perceived stressfulness, the supporting resources, defences and the coping mechanism of the individual[29] also influence the relationship between life events and psychiatric morbidity. It must be emphasized that in the sample studied, the majority did not report personal life events. Considering the fact that 60.0% perceived the job as stressful and 64.2% had no job satisfaction, it may be considered that for many, the work environment itself was stressful.

Stepwise multiple regression reveals that educational level, job satisfaction, perceived stress and stressful life events were independent determinants of psychiatric morbidity. The level of education is associated with various factors like job title, economic status and the combined effects of these can influence mental status. Further studies are required to elucidate whether modifying the level of education will be of any help to relieve psychiatric morbidity. Job satisfaction may have many influencing factors and warrants a focused study. The results also suggest that there is scope for psychiatric intervention for the management of perceived stress and for effective coping with stressful life events. This may have both therapeutic and preventive value.

This study has a few limitations: 1) a structured standardised diagnostic instrument was not used for the diagnostic process; 2) job attributes were evaluated as dichotomous variables whereas scales measuring them in a continuous spectrum would have been more appropriate, 3) results may not be extrapolatable to other types of industry, 4) physical and chemical parameters of an industrial environment were not studied. In summary, a significant proportion of the industrial population has psychiatric morbidity. Substance abuse, depression, anxiety and sleep disorders are the common psychiatric problems. The incidence of psychiatric caseness was significantly higher in persons with lower education, lower income, poor job satisfaction, stressful life events and those who perceived stress in the work environment.

## References

[1] World Health Organisation (WHO) (1986). Epidemiology of Occupational Health, Assessment of Occupational Health.

[2] Schottenfeld R, Levy BS, Wegman DH (1995). Psychiatric disorders. Occupational Health-Recognizing and Preventing Work-related Disease.

[3] Park K (1994). Occupational health. Park's Textbook of Preventive and Social Medicine.

[4] Karasek R, Theorell T (1992). Healthy work-Stress, Productivity and the reconstruction of working life.

[5] Sauter SL, Murphy LR, Hurrell JJ (1990). Prevention of work related psychological disorders. A national strategy proposed by the National Institute for Occupational Safety and Health (NIOSH). Am Psychol.

[6] Sadock VA, Kaplan HI, Sadock BJ (1995). Other clinical condition that may be a focus of clinical attention. Comprehensive Textbook of Psychiatry.

[7] Hensing G, Alexanderson K, Akerlind I, Bjurulf P (1995). Sick leave due to minor psychiatric morbidity: Role of sex integration. Soc Psychiatry Psychiatr Epidemiol.

[8] Baker DB, Karasek RA, Levy BS, Wegman DH (1995). Occupational stress. Occupational health - Recognizing and preventing work-related disease.

[9] Tar-Ching AW (1989). Industrial accidents under reported and not improving. Br Med J.

[10] Ganguli HC (1968). Prevalence of psychological disorders in an Indian industrial population Part I, II and III. Indian J Med Res.

[11] Bhaskaran K, Seth RC, Yadav SN (1970). Migration and mental ill health in industry. Indian J Psychiatry.

[12] Kar N, Dutta S, Patnaik S (2002). Mental health in an Indian industrial population: Screening for psychiatric symptoms. Indian J Occup Environ Med.

[13] Reddy MV, Chandrashekar CR (1998). Prevalence of mental and behavioural disorders in India: A meta-analysis. Indian J Psychiatry.

[14] Glozier N (2002). Mental ill health and fitness for work. Occup Environ Med.

[15] Kar N (2003). Mental ill health in workers: Observations from a few Indian populations. Occup Environ Med.

[16] Singh G, Kaur D, Kaur H (1984). Presumptive stressful life event scale: A new stressful life event scale for use in India. Indian J Psychiatry.

[17] Goldberg D, William P (1988). A user's Guide to the General Health Questionnaire (GHQ).

[18] Jacob KS, Bhugra D, Mann AH (1997). General health questionnaire-12: Psychometric properties and factor structure among Indian women living in United Kingdom. Indian J Psychiatry.

[19] Shamasundar C, Murthy SK, Prakash OM, Prabhakar N, Krishna DK (1986). Psychiatric morbidity in a general practice in an Indian city. Br Med J (Clin Res Ed).

[20] Coombs RH (1997). Drug-Impaired Professionals.

[21] World Health Organisation (WHO) (1992). The ICD-10 Classification of mental and behavioural disorders. Diagnostic criteria for research.

[22] Akerstedt T, Knutsson A, Levy BS, Wegman DH (1995). Shift work. Occupational health - Recognizing and preventing work-related disease.

[23] Kar N, Dutta S, Kar GC (1995). Injuries of shift work. Industrial Psychiatry J.

[24] Hollingstead AB, Redlich FC (1958). Social class and mental illness.

[25] Cooper CL (1983). Identifying stress at work: Recent research developments. J Psychosomat Res.

[26] Kawakami N, Araki S, Kawashima M (1990). Effects of job stress on occurrence of major depression in Japanese industry: A case-control study nested in a Cohort study. J Occup Med.

[27] Romanov K, Appelberg K, Honkasalo ML, Koskenvuo M (1996). Recent interpersonal conflict at work and psychiatric morbidity: A prospective study of 15,530 employees aged 24-64. J Psychosomat Res.

[28] Kessler RC, Kaplan HI, Sadock BJ (1995). Sociology and psychiatry. Comprehensive textbook of psychiatry.

[29] Kaplan H, Sadock B (1991). Synopsis of psychiatry: Behavioural science and clinical psychiatry.

